# Patient and Community Organization Perspectives on Accessing Social Resources from the Emergency Department: A Qualitative Study

**DOI:** 10.5811/westjem.2020.3.45932

**Published:** 2020-06-24

**Authors:** Margaret E. Samuels-Kalow, Melanie F. Molina, Gia E. Ciccolo, Alexa Curt, Emily C. Cleveland Manchanda, Nicole C. de Paz, Carlos A. Camargo

**Affiliations:** * Massachusetts General Hospital, Harvard Medical School, Department of Emergency Medicine, Boston, Massachusetts; † Boston Children’s Hospital, Harvard Medical School, Division of General Pediatrics, Boston, Massachusetts

## Abstract

**Introduction:**

Social risks adversely affect health and are associated with increased healthcare utilization and costs. Emergency department (ED) patients have high rates of social risk; however, little is known about best practices for ED-based screening or linkage to community resources. We examined the perspectives of patients and community organizations regarding social risk screening and linkage from the ED.

**Methods:**

Qualitative interviews were conducted with a purposive sample of ED patients and local community organization staff. Participants completed a brief demographic survey, health literacy assessment, and qualitative interview focused on barriers/facilitators to social risk screening in the ED, and ideas for screening and linkage interventions in the ED. Interviews were conducted in English or Spanish, recorded, transcribed, and coded. Themes were identified by consensus.

**Results:**

We conducted 22 interviews with 16 patients and six community organization staff. Three categories of themes emerged. The first related to the importance of social risk screening in the ED. The second category encompassed challenges regarding screening and linkage, including fear, mistrust, transmission of accurate information, and time/resource constraints. The third category included suggestions for improvement and program development. Patients had varied preferences for verbal vs electronic strategies for screening. Community organization staff emphasized resource scarcity and multimodal communication strategies.

**Conclusion:**

The development of flexible, multimodal, social risk screening tools, and the creation and maintenance of an accurate database of local resources, are strategies that may facilitate improved identification of social risk and successful linkage to available community resources.

## INTRODUCTION

Emergency department (ED) patients have high rates of non-medical but health-related needs, including both food and housing instability.^1^ A number of different terms have been used to describe these individual-level, adverse social determinants of health. For the purposes of this paper, we will term these “specific adverse social conditions that are associated with poor health” as *social risk*.^2^ Social risks are associated with higher disease prevalence, worse disease control, and resultant patterns of hospital utilization that include increased ED utilization^3^,^4^ and higher healthcare costs.^5^ Recent policy changes, including the creation of accountable care organization (ACO) models, are increasing emphasis on social risk by mandating screening and allowing organizations to use payments to address social risk.^6^ Both the Accountable Health Communities project^7^ and several Medicaid ACO demonstration studies are currently studying strategies for social risk screening and referral to community resources.^8^,^9^

Thus far, most of the policy emphasis has been on screening and linkage to resources in the primary care setting, and existing programs have demonstrated significant challenges in improving health outcomes and reducing healthcare utilization. A recent large evaluation of a phone-based screening and navigation program found only small decreases in healthcare utilization in the intervention group.^10^ Interventions directly targeting community-based organizations have also had little impact on healthcare utilization.^11^ Other studies, including one in the ED, have used a help-desk model of undergraduate volunteer navigators, and found no difference in ED utilization or need resolution.^12^ Similar interventions requiring significant staffing, potentially including community health workers, have shown promise but may be more challenging to scale outside of academic centers.^13^,^14^

With the increasing emphasis on social risk screening in novel payment models such as the ACO, and the high prevalence of social risk in ED patients who may not be accessing primary care, institutions are beginning to pilot screening and linkage interventions in the ED.^15^ However, little is known about best practices for linking ED patients to community resources in a time- and staff-efficient manner that is both useful for patients and feasible for the receiving community organizations.^6^ In particular, the perspectives and preferences of ED patients and receiving community organizations have not been well described. Therefore, the goal of this study was to examine the perspectives of patients and community organizations regarding social risk screening and linkage from the ED.

## METHODS

### Study Design and Setting

We conducted an in-depth, qualitative interview study with a purposively selected sample of ED patients as well as staff from regional community organizations, including homeless shelters and food banks. We chose in-depth interviews to identify the range of opinions regarding ED-based, social risk screening and linkage programs and elucidate new ideas and concepts.^16^ As is standard in qualitative studies, we used purposive sampling to “select representatives from various cross-cutting status positions that are relevant to individual experiences and beliefs with respect to the topic at hand”^16^ and concluded when thematic saturation was reached, or no new information was provided on the topic of interest in each of the prespecified status positions or groups.

**Population Health Research Capsule**

What do we already know about this issue?

*Emergency Department (ED) patients have high rates of social risk, however little is known about best practices for ED-based screening or linkage to community resources*.

What was the research question?

*To examine the perspectives of patients and community organizations regarding social risk screening and linkage from the ED*.

What was the major finding of the study?

*Participants felt it was important to screen, were concerned about linkage, and provided suggestions for program development*.

How does this improve population health?

*Participants highlighted the potential of ED social risk screening to reach vulnerable patients, identified barriers, and generated ideas for improvement to optimize population health*.

Qualitative interview guides were developed by the study team, piloted, and then refined. Interviewers received qualitative methods training, and direct feedback following each round of interviews. Interviews were conducted until thematic saturation was reached. This was deemed to have occurred when subsequent interviews failed to provide new information in each of the predefined groups (English speakers, Spanish speakers, community organization staff).^16^ This study was approved by the Partners Healthcare institutional review board.

### Selection of Participants and Participant Categories

Patients were recruited from a large, urban, academic ED. Bilingual research assistants (RA) screened patients for eligibility. Eligible patients included adults or parents/guardians of pediatric patients, who spoke either English or Spanish and were expected by the clinical team to be discharged at the conclusion of their ED visit. Patients on an involuntary mental health hold or with active intoxication were excluded. Community organizations were identified through hospital directories, social work, and use of the United Way 211 website. Community organizational staff were contacted for participation using a standard email. Community organization interviews were conducted at the organization and in English. (Please see Methodological Appendix for more details).

### Measurements

Patient participants completed a brief demographic survey and a health literacy assessment (Newest Vital Sign)^17^,^18^ in either English or Spanish. Qualitative interviews focused on barriers and facilitators to social risk screening in the ED, choice of ED as a care location, and ideas for screening and linkage interventions in the ED (Table 1).

All patient participants received an ED resources sheet outlining community resources for social risks. Community organization participants completed a brief demographic survey and a qualitative interview in English covering the same domains, with slightly modified questions (Table 2).

### Analysis

All interviews were recorded and professionally transcribed. A coding tree was developed based on the interview guide, and refined with input from the entire team. Transcripts were coded by two independent members of the research staff, with differences resolved by team consensus. Spanish-language transcripts were coded by bilingual study team members, and Spanish-language quotes are presented in the manuscript verbatim with translations from the study team following. Coding and theme development were ongoing throughout the study process, with adjustment of the coding tree and interview guide as themes emerged. Analyses used a modified grounded theory framework.^19^ Interviews were conducted until thematic saturation, as identified by consensus, was reached among patients within each predefined group (English speakers, Spanish speakers, community organization staff).

## RESULTS

### Characteristics of Study Subjects

Twenty-two interviews were conducted, of which 16 were with patients and six were with community organization staff. Of the patient participants, 11 (69%) spoke English and five (31%) spoke Spanish. Eleven (69%) had adequate health literacy and five (31%) had limited health literacy. Table 3 summarizes the demographic characteristics for patient participants. Of the six community organization participants, positions ranged from community health worker to director of a community health coalition, with a range of 3–31 years of experience in their respective sectors.

### Main Results

Three categories of themes emerged. The first related to drivers of ED utilization and emphasized the importance of screening for social risk in the ED. This category included themes around challenges accessing primary care providers (PCP) and inconsistent screening at PCP offices. The second category related to challenges around screening and linkage to community resources. Themes in this category included concerns around fear and mistrust of the healthcare system, the collection, maintenance, and transmission of accurate information, as well as time and resource constraints. In the third category, both patients and community organization staff provided suggestions for improvement and program development.

In each of the three categories, there were few differences in perspectives between patients by language or health literacy. Overall, resource scarcity was emphasized more by community organization staff. Staff also highlighted the importance of bidirectional and multimodal communication strategies with users of their services, whereas patients had more variation in preferences for specific verbal vs electronic strategies.

### Challenges in Primary Care Access and the Resulting Importance of ED Screening

Many patients reported challenges accessing primary care related to timing, cost, and availability of appointments, although a few patients highlighted the potential of the PCP in addressing social risk (Table 4).

Patient participants reported a broad range of experiences with social risk screening in the primary care setting. Some participants reported being screened for specific social risks in the clinic setting either verbally [“Somebody asked me…It was a type of questionnaire like this if I will need help with the utilities so I just answered yes” (limited literacy) or electronically [“Well when you go there and you check in they just give you a little tablet with some questions and then you answer the questions with what type of resources you think you will need. So I answer through that” (limited literacy)]. Others reported seeing posters with information but had not been asked directly.

Community organization staff also emphasized the importance of the ED as a screening location: “It’s like you’re often seeing people at a really critical time and they may be more down and out…if they had a plan for access to counseling for their mental health needs and potential medication and stuff like that. And direction to food and shelter. And having that all laid out and have someone as a point of contact for them even if it’s only during business hours or whatever just having-- I mean, caseworkers exist and all that stuff, but having more of that through the hospital could be good” (community organization staff).

### Challenges Around Screening and Linkage

#### Fear and Mistrust

Community organization staff and patient participants alike raised concerns around trust in both the healthcare and social services systems, in addition to fear of using resources. Participants reported concerns about stigmatization: “I would definitely say social discrimination is a huge barrier as well in many ways…People with different diagnoses might have barriers as well like substance use disorders, getting housing might be difficult if you have any kind of criminal record” (community organization staff). In addition, several staff participants discussed barriers related to recent policy changes, particularly for immigrants: “I think fear, immigration fear is a giant, giant concern right now that we see people aren’t coming out for services and they’re not signing up for services that they might be eligible for. So the political climate has really been an issue” (community organization staff).

Although most participants focused on recipient mistrust, one patient reported concerns about whether the health system or social service providers could trust the patients, who might be lying to access resources: “Por lo que--Tú sabes que muchas veces la gente puede omitir información, o muchas veces mienten para tener o conseguir más…Entonces no sé de qué manera podría llegar, o de qué manera--O sea, es una simple encuesta, yo sé; pero de qué manera comprobar de lo que están diciendo sea verídico” [*So—You know that many times people can omit information, or many times they lie to have or get more…So I don’t know in what way it could get to, or in what way—That is, it’s a simple survey, I know; but in what way to verify that what they are saying is truthful*] (adequate literacy).

#### Collection, Maintenance, and Transmission of Accurate Information

Patient participants emphasized the importance of providing accurate information about resources: “Yo pienso que muchas veces las informaciones son ya un gran recurso, es decir, existen estos recursos que se pueden utilizar en ciertas condiciones, porque hay mucha gente como yo que no la conoce toda, y por eso muchas veces uno se encuentra en grandes dificultades” [*I think that many times the information is already a great resource, that is to say, these resources exist that can be utilized in certain conditions, because there are many people like myself that do not know it all [the information], and because of that many times one finds oneself in great difficulties*] (limited literacy). The importance of accurate information was repeatedly emphasized with regard to resource availability and cost: “Providing as much information as possible so people know what services are there and know that it’s not going to cost them anything, or at least have an idea of what it would if there was a cost” (adequate literacy). Community organization staff discussed the importance of establishing accurate resource databases and being very clear with patients about the type of help that is available.

Patients discussed the challenges of obtaining information from hospital posters. In particular, they highlighted difficulties with remembering or retaining the information: “It’s just like it’s not really pamphlets, so it’s not really anything that I can take with me…It’s in the bathroom when you’re sitting in a stall and it says, ‘Are you in danger?’…I mean, so the stuff is there, but unless you’re writing it down or you take a picture of it with your phone…I might see it and go like, ‘Oh, wow. I would really like to do that,’ but remembering to take a picture, remembering to grab that information could be hard” (adequate literacy). Others identified challenges understanding information when it was provided only in English: “No. Yo no he visto, es que muchas veces se llega con tanta preocupación y la otra cosa es que podrían estar en inglés, no comprendo el inglés y bueno” [*No. I have not seen, it’s that many times you arrive with a lot of worry and the other thing is that they could be in English, I don’t understand English, and well…*] (limited literacy).

#### Time and Resource Constraints; Complexity of Navigation

Community organization staff, in particular, spoke repeatedly about the challenges of navigating the complex social service infrastructure to obtain resources: “Because I know someone came in looking for a detox bed and I tried to sit down with him and talk to him but I had no idea where to start. And I called all these different centers and they had all these different policies. …I don’t know how to navigate this? I’m very literate on a computer. I know how to use a computer. I’m very comfortable making phone calls. I’m a fluent English speaker and I still can’t figure this out. So I definitely had just a moment of frustration with how complex the system is and if there was a way to get other information really accessible, I think that that would be amazing and really change how things were working” (community organization staff). Others emphasized the importance of knowing what resources are actually available: “They may have a five-year waiting list. And the provider in the ER may not know that. And it’s hard to know what all the capacity is for a different agency” (community organization staff).

Community organization staff, as well as some patients, referenced time and attention constraints within the ED visit as potential barriers to screening effectively: “I think, yeah, I don’t at all disagree with you but I also think people in the emergency room, by the time they’ve been in the emergency room and seen a doctor can be so ready to leave but they’re not going to sit around and wait for a social worker to come down and talk to them even if that would be great. I’ve seen it in the ER a lot of times, that people are just like, ‘I’m out of here. I’m not sticking around. I’m not interested in going through my complex care plan with you. I just want to leave’” (community organization staff).

### Improvements and Next Steps

When asked about new tools for screening, participants reported mixed preferences for verbal vs electronic screening: “because they’re personal questions. Someone might feel more comfortable answering them through text, but at the same time they feel like they’re very personal questions, so it feels weird answering them through text. So kind of the same answer for both. Opposite reasons” (adequate literacy).

Regarding novel strategies for linkage, participants emphasized the importance of having a centralized directory of resources (Table 4) and being able to access information easily on-demand across modalities: “I think it would be really good if there were multiple points of entry and multiple points of access. So I don’t think it -- I think if it’s not an either -- or but if it’s somehow both. That you can have access to resources right then but then there’s also ways to engage at later points that are really accessible maybe through texting. I think that that’s awesome” (community organization staff).

### Patients Compared to Community Organizations

As compared to the patient participants, community organization staff were more skeptical about resource availability and more focused on bidirectional and multimodal communication. Community organization staff emphasized the challenges around accessing scarce social resources: “I think you just should be careful about offering housing resources because waitlists are 5, 10 years long. I just talked to somebody last week. I was doing an interview myself with someone last week who works with housing issues and she said she even has somebody on the emergency housing list that’s been on it for five years. So to be offering. I think you have to be careful when you say do you want resources with housing because people will jump on that because there’s really not much out there. So I think not over-promising” (community organization staff).

More than the patient participants, staff focused on the follow-up for positive screens: “I think it’s great that people are asking these questions because they’re so important. I would just want to make sure that they’re doing it for a reason and that it’s not just out there in the atmosphere. That somebody actually follows up and goes over the answers with them if their answers show that it needs follow up” (community organization staff). Finally, staff were also more concerned about the loss of information in transfer and translation between hospital providers and patients, emphasizing the importance of personal communication and the direct transmission of information: “So if you have somebody who could make that connection and connect patients, do a warm handoff, what we say warm handoff to resources. Sometimes, in the healthcare system, we’re used to like, ‘Oh, here is the sheet. There you go. Oh, it’s translated,’ but it could not be clear in that language…So there could be very simple thing that people don’t know about that you can help them brainstorm how to access that resource. And they just sometimes won’t unless somebody is there cheerleading them to do that” (community organizer staff).

## DISCUSSION

Screening for social risk among ED patients is an area of increasing interest across many healthcare systems. In this study, we sought to better understand the facilitators and barriers to social risk screening in the ED, as well as opportunities to develop mechanisms to link ED patients with social risks to community organizations. Through in-depth interviews, ED patients and community organization staff confirmed the importance of social risk screening in the ED, while also identifying several important barriers to screening and referral. Participants also identified strategies for improvement.

Overall, study participants felt that ED screening for social risk was important and valuable. However, they also raised concerns around fear and mistrust – particularly in the current political environment. Establishment of systems for social risk screening in the ED must take into consideration the particular concerns and needs of each hospital’s patient population, including fears of stigmatization based on social risk. To address concerns about fear and mistrust, programs must take appropriate measures to ensure secure collection and storage of patients’ social risk information and provide transparency around how and with whom the information is shared, particularly for immigrants and other vulnerable groups.

Patient participants had mixed preferences for the modality of screening, with some strongly preferring verbal and others recommending electronic. Given differing patient preferences about screening modalities, programs will need to consider their specific patient population to determine the acceptability of in-person vs technology-based screening. The development of multimodal, multilingual screening tools with systems that allow for flexibility even within a single healthcare facility may foster improved acceptance among both patients and healthcare providers.

Additional barriers to acceptable and efficient social risk screening in the ED identified in this study included time and resource constraints of both patients and providers, the collection and transmission of accurate information, and the complexity of the social service infrastructure into which patients are referred. While time and resource constraints for ED patients and staff can vary considerably across different care environments, the development of screening strategies that do not require clinical provider time and involvement may help increase feasibility and acceptability.

Both patients and community organization staff highlighted the importance of developing and maintaining a centralized resource directory in order to allow users to access accurate resource information at multiple times and in multiple ways. Compared to patient participants, community organization staff were more focused on the limitations of available resources and the importance of in-person navigational assistance to ensure that patients did not get lost in the transition from referral to resource. Interestingly, while community organization staff raised concern about the fear of resource utilization in relation to becoming a public charge, no patient participants identified this concern. However, we intentionally did not include any questions related to immigration status or citizenship in our study. Therefore, it is impossible to know whether the absence of this concern related to the public charge rule is a result of having interviewed only patients for whom this is not a concern, or whether they were reluctant to raise this concern with study staff.

In the setting of increasing policy emphasis on addressing social risk,^8^,^9^ a variety of screening and linkage programs have been developed. However, as mentioned previously, most have focused on screening in the primary care setting and many have struggled to successfully connect patients to resources. Notably, a recent large study of over 34,000 patients found that 53% screened positive, but only 10% were able to connect with resources to address their needs.^10^ Another study found that only 19% of patients with a health-related social need reported in the electronic health record had a documented referral placed.^20^ These studies further underscore the importance of developing interventions that not only identify social risk, but successfully link patients to adequate resources in their communities.

Our study’s participants also emphasized the need to create systems that establish true linkages between patients and community resources. Community organization staff in particular highlighted the need for personalized, repeated contact between individuals with social risk and those with the knowledge of how support systems operate in that individual community. Developing a robust system for linkage to community resources must incorporate accurate, timely, and confidential information-sharing between programs and community organizations. Ideally, such a system would be supported by a centralized resource directory. While further research is needed to understand optimal linkage strategies from the ED (eg, direct information provision vs hands-on navigation, follow-up mechanisms to ensure linkage and assist with troubleshooting, etc.), ED-based, social risk screening and linkage programs should be built on a foundation of understanding local resource availability and community organization capacity.

## LIMITATIONS

As with all qualitative studies, this work is hypothesis generating, not hypothesis testing. Due to staff capabilities, we were only able to enroll in English and Spanish, and, reflecting underlying demographics of hospital usage, we had limited racial diversity in our sample. In addition, we deliberately did not screen for social risk in our sample as we did not want to bias participant opinions by the use of one particular tool. As a result, however, we did not know the social risk status of the patient participants and thus cannot know how that might have affected their perspectives on screening and linkage.

## CONCLUSION

In this qualitative study, we examined perspectives of both ED patients and community organizations regarding ED-based screening for social risk and linkage to community resources. Participants highlighted the potential of ED-based social risk screening to reach vulnerable patients, who may otherwise not be identified through PCP-based screening programs. They also highlighted important barriers to successful screening and linkage, and generated ideas for optimizing such programs. The development of flexible, multimodal screening tools as well as the creation and maintenance of an accurate centralized database of local resources may facilitate improved identification of vulnerable patients and successful linkage to available community resources.

## Supplementary Information



## Figures and Tables

**Table 1:** Patient interview questions regarding screening for social risk in the emergency department.

Topic	Domain	Sample questions
Social risk screening	Experience	Would you like to share anything else about your experience with the survey we just walked through?
	Barriers	Were there parts that you yourself or others may not want to answer?
	Facilitators	That you found or others would find hard to answer? That you found or others would find easy to answer?
	Suggestions for improving	How could we improve the experience answering these questions for you or others? How can we make these questions more useful to you/others?
Resource linkage	Experience	Now we are going to switch gears a little and talk about your personal experience here in the ED: Have you ever been given information about additional resources from the ED (for example, housing assistance from social work)? Have you ever been given information about additional resources from your primary care provider?
	Barriers	What might make it hard to access those resources? (probe for ED− and PCP− provided resources
	Facilitators	What might help you access those resources? (probe for ED− and PCP− provided resources)
	Suggestions for improving	How could ED staff do a better job connecting people in the ED with community resources?
Choice of ED as care location	Barriers	Do you have a primary doctor or clinic? Is there anything that might make it hard for you or others to go there when you need care?
	Facilitators	Is there anything that makes it easier to go there when you or others need care?
	Decision making	Tell me about why you chose to come to this location today? (not reason for seeking care/but why this location) Did you seek care anywhere else for this problem before this visit?
	Barriers to ED use	What makes it hard for you or others to receive care in the ED? (probe for domains of social risk)
	Facilitators of ED use	What makes it easier for you or others to receive care in the ED? (probe for domains of social risk)

*ED*, emergency department; *PCP*, primary care provider.

**Table 2:** Community organization interviews regarding how emergency department staff connects patients to community resources.

Topic	Domain	Sample questions
Social risk screening	Suggestions for improving	How can we improve patients’ experience answering these questions? What information would be helpful for you to get about patients referred from the ED?
Resource linkage	Experience	Can you tell me about how people get referred to your organization? Specifically, from the healthcare system? From the ED?
	Barriers	What challenges do patients face accessing community resources?
	Facilitators	What makes it easier for patients to access community resources?
	Suggestions for improving	How could ED staff do a better job connecting people in the ED with community resources?
	Logistics	What would be the best way to connect a patient with your organization? Please tell me about your intake for new participants. Is there anyone else you think we should talk with about this?

*ED*, emergency department.

**Table 3:** Demographics of patient participants.

	Primary language		Total n (%)
Age†	English	Spanish	
			
30–40	8	2	10 (67)
41–50	3	1	4 (27)
51+	0	1	1 (6)
Gender			
Male	3	0	3 (19)
Female	9	4	13 (81)
Race/Ethnicity			
Hispanic	1	4	5 (32)
Non-Hispanic White	8	0	8 (50)
Non-Hispanic Black	1	0	1 (6)
Asian	1	0	1(6)
Non-Hispanic Other	1	0	1 (6)
Insurance			
Private	8	0	8 (50)
Public/ state	4	4	8 (50)
Total			16 (100)

† One participant preferred not to not provide an age.

**Table 4:** Themes and representative quotes from patients facing social risks and community organizers regarding access to aid.

Category	Theme	Patient participant	Community organization participant
Importance of ED screening	PCP access	Their hours. They close at three …I get out of work at 3:00. They get out of school at 3:00. You can't see them during the week. And they only see very sick babies on the weekends. So basically, in order to go with these kids for anything, I need to take a day off from work. They need to take time off from school, which is kind of not right. (Adequate literacy)	
	Inconsistent PCP screening	I see them all over the walls. Posted. Oh, I need help with…I don’t recall being asked directly, I guess…If you need help to quit smoking. For domestic violence or something, you can call this number. Bunch of random stuff. (Adequate literacy)	
Challenges around screening and linkage	Fear and trust	Not knowing where and who to go to and even being afraid of asking questions mostly. (Adequate literacy)	Specifically, it’s the fear of receiving any help from anybody if you’re undocumented. … It’s just the fear of what it is and how much information do I have to provide in order to receive the benefits…especially with the fear of immigration and deportations. Even people who are documented, who are in the path to receiving green cards, and who are eligible to receive green cards, they say, “No, thank you” because now there’s that fear that if I’m using public benefits, that public charge clause would apply.
	Collection, maintenance, and transmission of accurate information.	I think he was a social worker from the Massachusetts General Hospital. I don’t know. But he called me. Yeah, he called me and he spoke with me over the phone and just -- he give me all the information and I wrote it down. And also he said that he was going to send me a mail with resources. And I got that in the mail as well. So that was good. (Limited literacy) Just thinking back about my local hospital and how I’m pretty sure that they have pamphlets for any sort of needs or feeling endangered in any way, but they’re not really prominent in the areas. (Adequate literacy)	But if you don’t have access to a phone or Iinternet regularly, then keeping track in your head when all these different things are open and when you can go and get services I think is probably really challenging. But that is what we find is always the biggest issue is that just handing someone a pamphlet or handing somebody a phone number is not always very effective. I’m sure you’ve heard the term warm hand-off. I think those are far more successful. So when somebody is actually helping the patient make the connection and make sure it’s a referral that’s appropriate and works.
	Time and resource constraints	So I’d be like, “Hey I can’t pay for this. What do I need to do to get some help?” And then if they had all the information you needed. You’re good. But if they’re like, “Oh, you need this. You need three month’s worth of utility bills, your three months of pay stubs--” if you need a whole bunch of stuff to get it done then people are going to get frustrated. (Adequate literacy) I’ve been in the emergency room in more difficult situations and I probably wouldn’t be answering questions in that moment. Yeah. But definitely before or after. I don’t see why not. So maybe if you could get the contact information and then, just text them after. (Adequate literacy)	But there are almost 12,000 patients, and there’s me. And so I can’t talk to everybody. But I know where the people can get free clothes and food and there’s always help there for the basic, basic things. A lot of times that leads them to a little bit of disappointment when they think that you’re going to give them something, and they’re like “Oh, you’re just here to give me paper. I don’t need paper.”
Strategies for improvement	Modality (electronic vs. verbal)	Just because everyone’s on their phone all the time, and it’s probably a good way to get at people, and maybe it won’t make them nervous if they don’t have to answer face-to-face or be embarrassed (Adequate literacy) Talking is extremely easy. But for me to understand it’s good. I understand what it was you was asking (Limited literacy)	Somebody who could connect them to resources, who looks like them and speaks like them. I would say that that’s been not necessarily like all of that combined, but it has to be some kind of a connection because really we’re looking at a lot of mistrust between either for the healthcare system or well, just not a lot of trust. I mean, I think in person would be so much more effective. But I understand the cost of that is probably not something that people are willing to take on. I mean, I hope that an organization with the resources that engage has or other hospitals would move in that direction. I think texting is a really good start for it. Yeah. But I think that in-person follow-up is so much more effective.
	Centralized resource information and coordination	Like a centralized location or yeah, a resource area. You know what I mean? If there was a place that we could go where those questions were asked, like, “Are you struggling with homelessness? Are you struggling to provide food?” If there was a certain area or resource place, I think that would be good because, from my recollection, it’s just posters and things that I see and little pamphlets that are over here, but it’s kind of spread throughout the healthcare center. (Adequate health literacy)	If there is some way for the hospital or some organization the hospital is working with to hold all the knowledge of all the organizations in the city and be able to share that. And be able to be updated on what places have beds and what their hours are and when their hours change.
	On demand information and navigation	Well, I think because you’re worried about so much else going on, and then if you’re just getting a quick text message that here we can help you with something that’s troubling you so much. I mean, if somebody has no food they’re really going to be worried, or they’re about to lose their utilities, and so they could say I can get your text and give you an answer and help you. I think that’s extremely useful. (Adequate literacy)	It really just depends on the need of the patient because if they are in need, and you give them the information they’ll be grateful. But some people, for example, the elderly, or if they have some sort of disability, they might need the advocate to help them. So it really depends on the person. But if there is a way to text and be like, “Where can I go right now to get food?” And if there was an automatic response, “Where can I go right now to get food in 02116?” Or, whatever. I think that would be really cool. If there was just something that was some sort of computer system that could just generate responses to questions. And if patients could be educated about how that works before they leave the ED so that then they have that information on hand. That’s one thing that comes to mind.

*ED*, emergency department; *PCP*, primary care provider.
