# Supplementary material for: Patient and Community Organization Perspectives on Accessing Social Resources from the Emergency Department: A Qualitative Study
Source: West J Emerg Med. 2020 Jun 24;21(4):964–73. doi: 10.5811/westjem.2020.3.45932 (PMC7390556; doi:10.5811/westjem.2020.3.45932)
Supplement: Supplementary file 1 [file wjem-21-964-s001.docx]

**Methodological Appendix**

**COREQ Supplemental Information**

*Personal characteristics.* Interviews were conducted by Alexa Curt, BS, and Gia Ciccolo, MPH, clinical research coordinators at Massachusetts General Hospital (MGH). Both interviewers are female and bilingual (English/Spanish); their interest in the research topic was originated by working for Margaret Samuels-Kalow. They were trained to conduct in-depth interviews by Margaret Samuels-Kalow, MD MSHP, who had completed formal qualitative methods training as part of her MSHP degree.

*Relationship with participants.* Interviewers did not have an established relationship prior to conducting the interview. Participants knew that the goal of the study was to identify barriers and facilitators to connecting patients to resources from the emergency department (ED).

*Theoretical framework.* Analyses used a modified grounded theory framework to identify novel themes from interviews.

*Participant selection.* Research assistant (RA) shifts in the ED were balanced across time-of-day to reduce bias, and patient participants were selected based on eligibility criteria and purposive sampling to balance across language (English/Spanish). Patients were recruited face-to-face. Community organizations were identified through hospital directories, social work, and use of the United Way 211 website. Community organizational staff were contacted for participation using a standard email. Twenty-two subjects were included. We do not have data on how many people refused to participate.

*Setting.* The setting of the data collection was in the ED or in the workplace. There were no non-participants present. The demographics of the sample are shown in the main paper.

*Data collection.*  The interview guide is presented in the main paper. There were no repeat interviews; audio recording was used to collect the data, and field notes were made during/after the interviews and were used to refine the interview guide and begin the analysis. The interviews were 20-30 minutes long and data saturation is discussed in the manuscript. No transcripts were returned to participants.

*Data analysis.* The data was coded by 4 investigators: Melanie F. Molina, MD (bilingual), Gia E. Ciccolo, MPH (bilingual), Emily C. Cleveland Manchanda, MD, MPH (monolingual), Nicole C. de Paz, MD (bilingual). The coding tree is presented below. Themes were derived from the data. NVivo software was used for data management. Participants did not provide feedback on the findings.

*Reporting.* Quotations were presented in the manuscript and identified by category of speaker. Data, findings, major and minor themes are presented in the main paper.

*Coding tree.*

1. PCP insufficient
   1. Not available
   2. Not enough
   3. Didn’t solicit resources
2. Resources complex and limited
   1. Resources are changing/what is available now/keeping up to date/knowledge of resources
   2. What can we actually offer/scarcity of resources
   3. Information gets lost/warm handoff/information transfer/closed loop
   4. Process of obtaining resources
3. Fear + trust (from both patient and community worker perspective)
   1. Embarrassment
   2. Public charge
   3. Secondary gain + lying
   4. Fear
   5. Trust/ rapport
4. Understanding and information transfer
   1. Text v. verbal
   2. Literacy
   3. Language
   4. Tech fluency, phone access, internet access
   5. Age
   6. Cultural sensitivity
5. Time constraints
   1. Patients have other competing needs/wanting to leave/distractions by acuity
   2. ED time constraints
   3. ED navigator capacity
6. Improvements
   1. Centralized resource place
   2. On demand info
